# Intraluminal washout in rectal and sigmoid colon cancer surgeries with double‐stapling technique anastomosis: A single‐institution prospective study

**DOI:** 10.1002/ags3.12851

**Published:** 2024-08-30

**Authors:** Shinji Furuya, Kensuke Shiraishi, Hiroki Shimizu, Koichi Takiguchi, Makoto Sudo, Akaike Hidenori, Yoshihiko Kawaguchi, Hidetake Amemiya, Tetsuo Kondo, Daisuke Ichikawa

**Affiliations:** ^1^ First Department of Surgery, Faculty of Medicine University of Yamanashi Chuo Japan; ^2^ Division of Digestive Surgery, Department of Surgery Kyoto Prefectural University of Medicine Kyoto Japan; ^3^ Department of Surgery Yamanashi Kosei Hospital Yamanashi Japan; ^4^ Department of Pathology, Faculty of Medicine University of Yamanashi Chuo Japan

**Keywords:** distal free margin, exfoliated cancer cells, intraluminal washout, rectal cancer, sigmoid colon cancer

## Abstract

**Aim:**

This study aimed to determine the necessity of intraluminal washout through cytological assessment to prevent implantation of exfoliated cancer cells (ECCs) in patients with rectal and sigmoid cancers.

**Methods:**

We studied 140 patients with either sigmoid or rectal cancer who underwent anastomosis surgery using a double‐stapling technique. An intraluminal washout sample was collected before and after irrigation with 1000, 1500, or 2000 mL of physiological saline or distilled water. Cytological assessments were conducted using the Papanicolaou classification system, where classes IV and V indicated positive cytological findings.

**Results:**

Initially, 46.4% of the patients (65 out of 140) had positive ECCs. Patients with cancer cells had a significantly shorter distal free margin (DM) from the tumor (*p* < 0.001). The length of the DM was significantly associated with the tumor distance from the anal verge (*p* < 0.001). After irrigation with 2000 mL, ECCs were found in only 7.3% of patients. Logistic regression analysis showed that DM (≤50 mm) and tumor size (≥50 mm) were independent risk factors for positive ECCs after intraluminal washout, regardless of the type of irrigation solution used.

**Conclusion:**

In patients with sigmoid colon cancer, adequate preoperative bowel preparation, a long DM, and a small tumor size, a 1000 mL intraluminal washout may be sufficient. By contrast, in patients with rectal cancer with a short DM and a large tumor size, a ≥2000 mL intraluminal washout is required. The different types of irrigation solution did not affect the ECCs. Large randomized controlled trials are required to confirm these results.

## Graphical abstract

This study investigated the need for intraluminal washout through cytological assessment to prevent the implantation of exfoliated cancer cells (ECCs) in patients with rectal and sigmoid cancers. Among 140 patients undergoing anastomosis surgery, initial findings revealed 46.4% with positive ECCs, associated with a shorter distal free margin (DM) and larger tumor size. However, irrigation with 2000 mL resulted in only 7.3% of patients showing ECCs, suggesting that the necessity of intraluminal washout depends on factors such as DM length and tumor size.

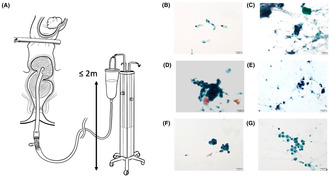

## 1 INTRODUCTION

Anastomotic recurrence at the site of surgery for colorectal cancer (CRC) is speculated to occur due to the implantation of detached malignant cells, although the exact mechanism remains unclear. This type of recurrence is considered a local recurrence and can significantly affect the quality of life and lead to serious outcomes.^1^ The local recurrence rate after rectal cancer surgery is approximately 5%–10%.^2^ Local recurrence remains a relatively common form of recurrence, along with liver metastasis, but is now surpassed by lung metastasis.^3^

Previous studies have reported the presence of intraluminal exfoliated cancer cells (ECCs) in patients with CRC.^4^ To prevent the spread of malignant cells within the bowel lumen, surgeons have avoided touching or manipulating tumors for 100 years.^5^, ^6^ The use of a standard circular stapler for anastomosis has been suggested to increase the accumulation of intraluminal ECCs at the anastomotic site, thereby increasing the risk of recurrence.^7^ Intraluminal irrigation is a widely used procedure globally to prevent ECCs transplantation in patients with CRC.

Several prospective clinical trials^8^ and meta‐analyses^9^ have evaluated the effectiveness of intraluminal lavage in preventing local recurrence, but its effectiveness remains controversial. A number of these studies have described the use of varying irrigation fluid volumes to efficiently remove ECCs. However, owing to the small sample sizes, no standard has yet been established for the appropriate volume or type of irrigation to use.^9^, ^10^, ^11^

Our institution, like many others, performs intraluminal washout for sigmoid colon resection with anastomosis by using a double‐stapling technique (DST), a technique similar to that used in rectal cancer surgery. This study aimed to determine the necessary and optimal methods (irrigation volume and solution type) for intraluminal washout based on clinicopathological factors, including tumor location.

## 2 METHODS

### 2.1 Patients

A total of 140 consecutive patients, comprising 91 men and 49 women with an average age of 68.9 years, who underwent sigmoidectomy or anterior resection for sigmoid colon cancer or rectal cancer at the University of Yamanashi (Yamanashi, Japan) between July 2018 and December 2022, were included in the study. Of these patients, 56 and 84 had sigmoid colon and rectal cancers, respectively.

### 2.2 Data collection

Clinicopathological findings were obtained from the hospital clinical records. The location of the tumor and position of its lower edge were determined using enema examination. According to the Japanese Classification of Colorectal Carcinomas, the rectum was divided into three sites: the rectosigmoid (RS), upper rectum (above the peritoneal reflection, Ra), and lower rectum (below the peritoneal reflection, Rb).^12^ Undifferentiated histological types include poorly differentiated mucinous adenocarcinomas and signet ring cell carcinomas. Local recurrence was defined as anastomotic, pelvic (including peritoneal dissemination confined to the pelvis), and lateral lymph node recurrence.

### 2.3 Preoperative bowel preparation

Our institute followed a standard preoperative bowel preparation procedure for patients without bowel obstruction. This procedure involved administering a combination of magnesium citrate (250 mL) and sodium picosulfate solution (0.75%, 10 mL) the day before surgery.

### 2.4 Measurement of distal free margin

The length of the distal free margin (DM) was determined based on the tumor location as follows: 10 cm at the sigmoid colon, 3 cm at the RS and Ra, and 2 cm at the Rb.^12^ During the surgery, the resected specimen was gently stretched and fixed with pins to measure the length of the DM.

### 2.5 Intraluminal washout and sample collection

**FIGURE 1:**
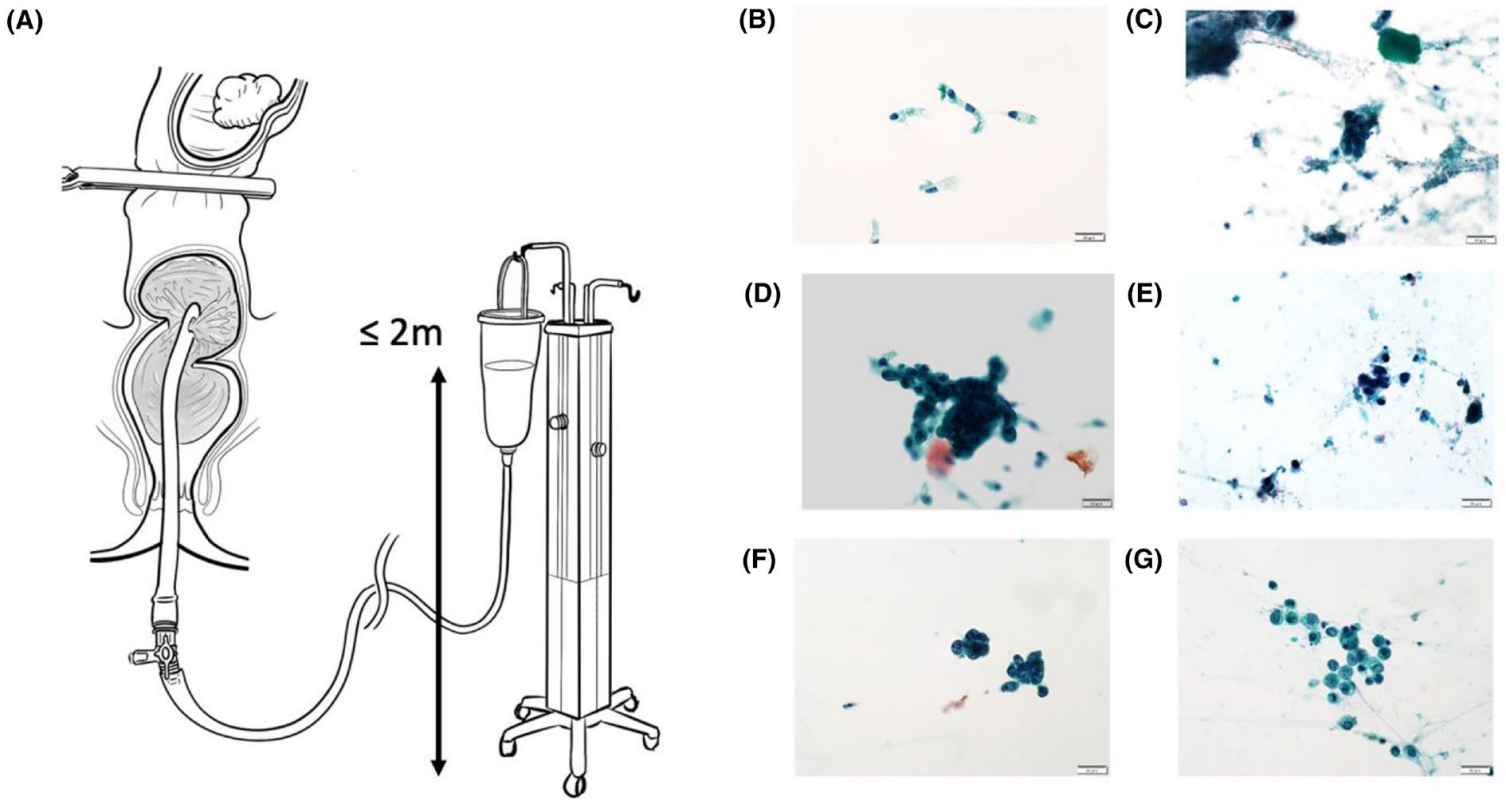
(A) Intraluminal washout procedure. The Nelaton catheter was inserted through the anus. The pressure for intraluminal washout was set at a maximum height of 2 meters. (B–G) The Papanicolaou classification system was used for cytological assessment of ECCs. (B) Class I (normal), (C) Class II (benign changes), (D) Class IIIa (mildly atypical cells), (E) Class IIIb (severe atypical cells), (F) Class IV (suspected malignancy), and (G) Class V (malignant).

Before dissection, the distal rectum was clamped to occlude the rectal stump from the tumor. Intraluminal washout was performed using a transanally inserted Nelaton catheter (8.5 mm in diameter) (Izumo Health Co., Ltd., Japan), with either physiological saline or distilled water. During washout, samples were collected at four time points: before washout and after irrigation with 500, 1000, and 2000 mL of physiological saline or distilled water (Figure 1A). Physiological saline was used as the irrigation solution from July 2018 to December 2020, and distilled water was used from January 2021 to December 2022. Samples were collected by injecting 20 mL of physiological saline solution into the lumen of the intestine.

### 2.6 Cytology

The collected samples were centrifuged at 3000 rpm for 5 min, and the resulting clots were examined using Papanicolaou staining. Two experienced cytotechnologists analyzed the stained samples to confirm the diagnosis, subsequently reviewed by a pathologist. The samples were classified according to the Papanicolaou classification system, with classes I, II, and III categorized as non‐malignant and classes IV and V as malignant (Figure 1B–G).

### 2.7 Statistical analysis

Statistical analyses were conducted using Prism (v.9, GraphPad Software, San Diego, CA). Comparisons were made using the chi‐square test or Fisher's exact test and the Mann–Whitney *U* test, as appropriate. Logistic regression analysis was performed to determine risk factors for positive EECs at each washout point. Statistical significance was set at *p* ≤ 0.05.

## 3 RESULTS

Table 1 shows the clinicopathological characteristics of the study participants. Among the 84 patients diagnosed with rectal cancer, 75% (*n* = 63) received various forms of preoperative treatment. By contrast, only 20% (*n* = 11) of the 56 patients diagnosed with sigmoid colon cancer received preoperative treatment. With regard to the tumor's anal margin location in the rectal cancer group, 35 cases were located at the RS, 19 at the Ra, and 30 at the Rb. No significant differences were observed in preoperative bowel preparation, type of irrigation solution used, surgical approach, tumor size, histological type, or depth between the rectal and sigmoid colon cancer groups. Notably, the DMs were significantly shorter in patients with rectal cancer than in those with sigmoid colon cancer (*p* < 0.001).

**TABLE 1:** Characteristics of 140 patients with sigmoid colon and rectal cancers.

Characteristics of patients					
Variable	Level	All	Sigmoid colon	Rectum	*p*‐value
		*n* = 140	*n* = 56	*n* = 84	
Gender	Male	91	38	53	0.335
	Female	49	18	31	
Age	Years	68.9 ± 9.4	70.1 ± 8.2	68.0 ± 10.0	0.201
Pretreatment	Negative	32	11	21	0.547
	Positive	108	45	63	
Bowel prepartion	Normal	107	40	67	0.874
	Reduced or none	33	16	17	
Type of intraluminal washout	NS	65	23	42	0.748
	DW	75	33	42	
Surgcal approach	Laparotomy	6	4	2	0.218
	Laparoscopy (robot‐assisted)	134 (26)	52 (0)	82 (26)	
Distal‐free margin	cm	6.5 ± 5.4	10.7 ± 6.1	3.8 ± 2.2	<0.001
Tumor size	mm	43.6 ± 18.5	46.4 ± 18.6	41.8 ± 18.3	0.144
Histology classification	Differentiated	137	56	81	0.275
	Undifferentiated	3	0	3	
Depth of tumor	T1	23	6	17	0.090
	T2	19	4	15	
	T3	76	35	41	
	T4a	18	8	10	
	T4b	4	3	1	
Lymph node metastasis	Negative	89	34	56	0.957
	Positive	53	24	28	

**FIGURE 2:**
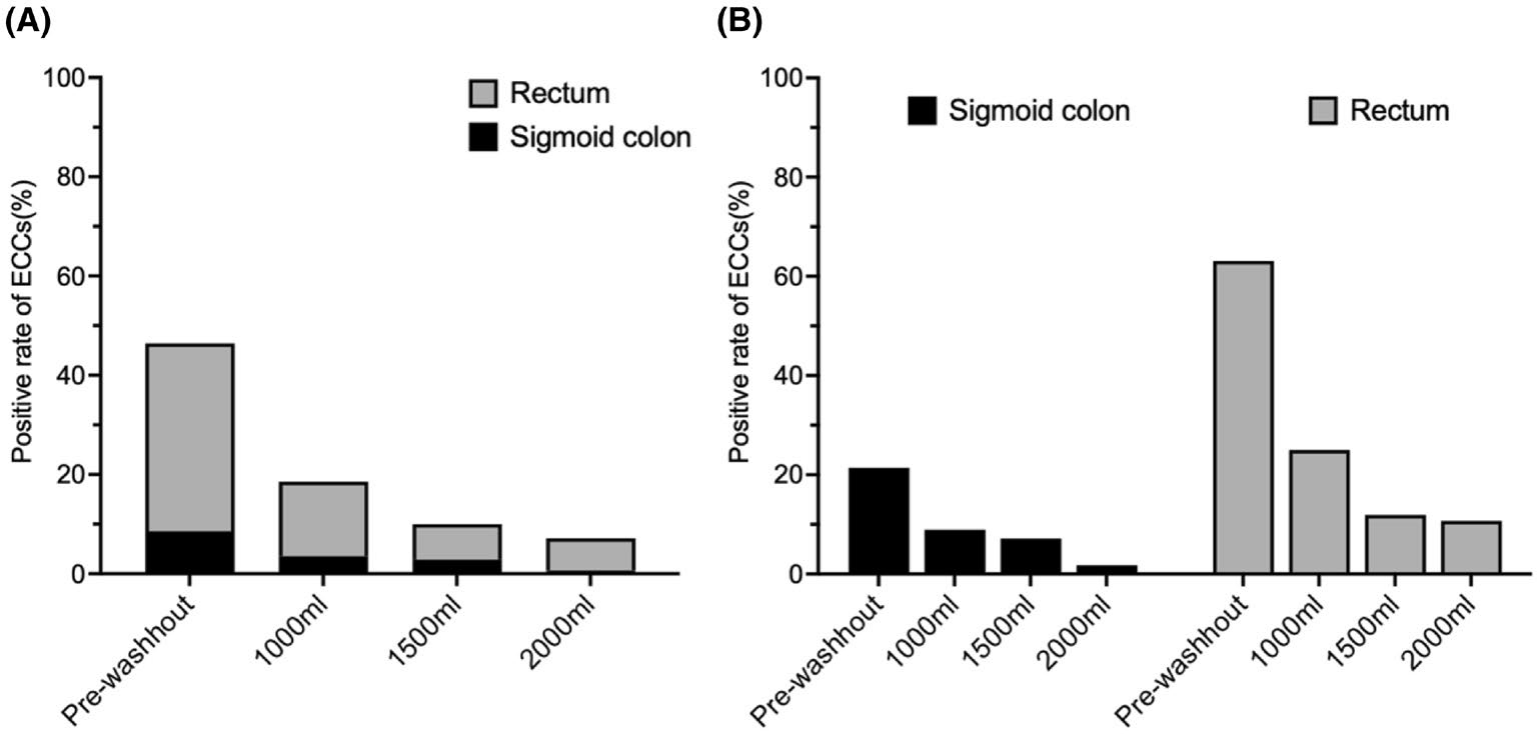
Positive rates of ECCs gradually decreased as the amount of irrigation increased. (A) All 140 patients. (B) Each tumor location (sigmoid colon and rectum).

Figure 2 illustrates the positive rates of ECCs detected in perfusate samples, collected at four time points. These rates are presented both as an overall percentage and categorized according to tumor‐occupied sites. Initially, 46.4% of all patients showed positive ECCs prior to the washout procedure. This rate progressively decreased with increasing perfusate volume; it was 18.5% after 1000 mL, 10.0% after 1500 mL, and finally, 7.1% following a 2000 mL washout. When analyzing data by tumor site, we observed differing patterns. For patients with sigmoid colon cancer, the positive rate of ECCs was 21% before washout, which decreased to 1.8% in one case after a 2000 mL washout. By contrast, 63% of patients with rectal cancer had ECCs before washout. Even after a 2000 mL washout, 10.7% of these patients still exhibited ECCs.

**FIGURE 3:**
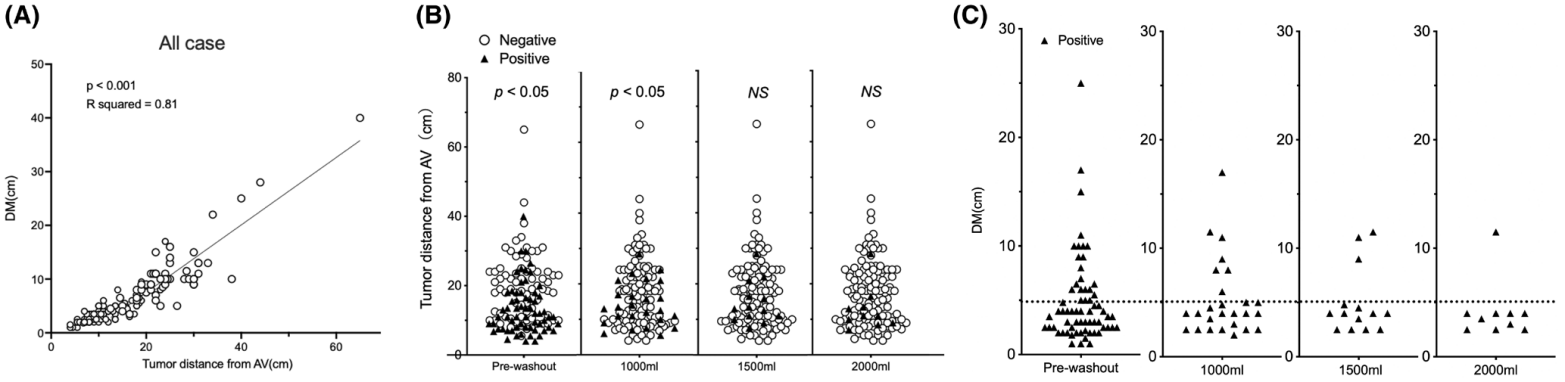
(A) Correlation plot between distal free margin (DM) and tumor distance from the anal verge (AV). (B) Plot of tumor distance from the anal verge and ECC determination at each intraluminal washout point. (C) Plot of DM and positive ECCs at each intraluminal washout point.

The relationship between the distance from the anal verge to the tumor and the DM is shown in Figure 3. Both variables were significantly and positively correlated (*p* < 0.001, *R* ^2^ = 0.01). Figure 3B shows the correlation between tumor distance from the anal verge and the detection of ECCs. A significant increase in positive ECCs was found in patients whose tumors were closer to the anal verge, both before and after the 1000 mL washout (*p* < 0.05). However, no significant difference was observed when comparing the results of the 1500 and 2000 mL washouts. The DM lengths in patients with ECCs before the washout procedure are shown in Figure 3C. Notably, one patient with sigmoid colon cancer who tested positive for ECCs following a 2000 mL washout had a DM length of 11.5 cm.

An analysis of the prewashout risk factors for the potential presence of ECCs was performed. In the univariate analysis, no significant differences were found in age, sex, type of irrigation solution, surgical approach, histological type, or the presence of lymph node metastasis. However, differences were observed in the presence of preoperative treatment, preoperative bowel pretreatment status, tumor location, DM, tumor size, and tumor depth at the four time points previously mentioned (Table 2). Multivariate analysis showed that a shorter DM (*p* < 0.03) and larger tumor size (*p* < 0.03) emerged as ultimate risk factors for positive ECCs after a 2000 mL irrigation (Table 3).

**TABLE 2:** Univariate analysis of clinicopathological risk factors for positive ECCs before intraluminal washout at each cleansing point.

Characteristics of patients		Univariate			
Variable	Level	*p*‐value (OR; 95%CI)			
		Pre‐washout	1000 mL	1500 mL	2000 mL
Gender	Male/female	0.425 (1.33; 0.662–2.68)	0.617 (0.791; 0.302–1.93)	0.596 (0.720; 0.189–2.29)	0.314 (0.441; 0.065–1.85)
Age (years)	70>/70≦	0.594 (1.20; 0.616–2.34)	0.305 (0.637; 0.264–1.50)	0.653 (1.29; 0.425–4.13)	0.575 (1.45; 0.397–5.91)
Pretreatment	Negative/positive	0.388 (1.42; 0.642–3.16)	0.003 (4.03; 1.61–10.1)	0.003 (5.67; 1.81–18.7)	0.009 (6.00; 1.60–25.0)
Bowel prepartion	Normal/reduced or none	0.598 (0.809; 0.363–1.77)	<0.001 (4.70; 1.90–11.8)	0.004 (5.39; 1.72–17.7)	0.002 (9.33; 2.42–45.6)
Type of intraluminal washout	NS/DW	0.689 (1.15; 0.588–2.24)	0.402 (0.694; 0.291–1.63)	0.400 (0.620; 0.194–1.88)	0.135 (0.345; 0.072–1.30)
Surgcal approach	Laparotomy/laparoscopy & robot‐assisted	0.858 (0.861; 0.154–4.80)	0.902 (1.15; 0.175–22.5)	0.584 (0.537; 0.078–10.7)	0.993 (3.43e+06; 4.92e‐61‐NA)
Lower edge of tumor	Sigmoid colon/rectum	<0.001 (6.27; 2.96–14.1)	0.021 (3.40; 1.28–10.7)	0.362 (1.76; 0.554–6.68)	0.078 (6.60; 1.19–123)
Distal‐free margin (mm)	50>/50≦	<0.001 (0.271; 0.133–0.541)	0.073 (0.444; 0.176–1.06)	0.031 (0.233; 0.051–0.787)	0.028 (0.095; 0.005–0.528)
Tumor size (mm)	50>/50≦	0.002 (3.00; 1.50–6.13)	<0.001 (7.22; 2.81–21.2)	0.005 (6.60; 1.94–30.3)	0.010 (15.9; 2.86–298)
Histology classification	Differentiated/undifferentiated	0.985 (6.97e+06; 1.78e‐41‐NA)	0.518 (2.24; 0.102–24.3)	0.992 (5.61e‐07; NA‐1.34e+72)	0.992 (8.11e‐07; NA‐1.68e+72)
Depth of tumor	T1‐3/T4	0.199 (1.83; 0.734–4.76)	0.024 (3.17; 1.13–8.59)	0.039 (3.56; 1.00–11.7)	0.211 (2.50; 0.506–9.91)
Lymph node metastasis	Negative/positive	0.126 (1.71; 0.862–3.43)	0.336 (1.53; 0.638–3.62)	0.685 (1.26; 0.393–3.85)	0.416 (1.71; 0.454–6.43)

**TABLE 3:** Multivariate analysis of clinicopathological risk factors for positive ECCs before intraluminal washout at each cleansing point.

Characteristics of patients		Multivariate			
Variable	Level	*p*‐value (OR; 95%CI)			
		Pre‐washout	1000 mL	1500 mL	2000 mL
Gender	Male/female				
Age (years)	70>/70≦				
PreTreatment	Negative/positive		NS	NS	NS
Bowel prepartion	Normal/reduced or none		0.045 (5.50; 1.05–31.5)	NS	NS
Type of intraluminal washout	NS/DW				
Surgcal approach	Laparotomy/laparoscopy & robot‐assisted				
Lower edge of tumor	Sigmoid colon/rectum	<0.001 (8.49; 2.88–28.1)	0.011 (1.47; 1.70–38.5)		NS
Distal‐free margin (mm)	50>/50≦	NS	NS	0.017 (0.154; 0.027–0.633)	0.023 (0.016; 0.001–0.361)
Tumor size (mm)	50>/50≦	<0.001 (5.75; 2.42–15.4)	<0.001 (7.78; 2.68–25.4)	0.037 (4.71; 1.18–23.8)	0.021 (13.7; 2.04–273)
Histology classification	Differentiated/undifferentiated				
Depth of tumor	T1‐3/T4		NS	NS	
Lymph node metastasis	Negative/positive				

## 4 DISCUSSION

The reported local recurrence rate after rectal cancer surgery ranges from 5% to 10%,^2^, ^3^ with the majority of cases occurring within the first 2 years after surgery. Specifically, 60%–80% recur in the first year and 90%–93% in the second year.^13^ In this study, local recurrence was observed in 1.4% of all cases (two of the 140 cases) despite the relatively short observation period (the median observation period was 919 days [range: 15–2022 days]). Both cases involved patients with rectal cancer. Specifically, local recurrence occurred in 2.4%, two of the 84 rectal cancer cases. These findings suggest that intraluminal washout may effectively reduce local recurrence rates. Of the two cases where local recurrence was observed, in one case, anastomosis site had positive ECCs after a 2000 mL intraluminal washout. In the other case, peritoneal dissemination confined to the pelvis with an abscess by microperforation at the tumor site preoperatively (data not shown).

In a previous study, the effectiveness of intraluminal washout with physiological saline in eliminating ECCs was evaluated in a limited number of patients with sigmoid colon and rectal cancers requiring a DST.^14^ The study reported that while patients with rectal cancer required a 1000 mL or more intraluminal washout, such volume may not be necessary for patients with sigmoid colon cancer. Building on these findings, this research expanded the sample size, increased the irrigation volume, included distilled water as an irrigation solution, and examined risk factors for positive ECCs. As a result, in patients with sigmoid colon cancer with adequate preoperative bowel preparation, a long DM, and a small tumor size, a 1000 mL intraluminal washout was considered sufficient. By contrast, in patients with rectal cancer with a short DM and a large tumor size, a 2000 mL or more intraluminal washout was necessary. This study is the first to investigate the volume of bowel irrigation and two types of irrigation solution in patients with sigmoid colon and rectal cancers who require a DST.

Intraluminal washout during rectal cancer surgery has been a longstanding surgical practice.^6^ Viable cancer cells, detached from the tumor, are present within the intestinal lumen adjacent to the tumor site.^4^, ^7^, ^11^, ^15^ Many surgeons have explored the advantages of intraluminal washout in preventing local recurrence.^16^ Examination of tissue collected with the DST circular stapler has demonstrated that intraluminal washout eliminates disseminated malignant cells.^10^, ^17^ However, the efficacy of intraluminal washout in eliminating ECCs is dependent on the irrigation volume. Previous reports have not consistently confirmed the disappearance of malignant cells in all patients, even with an irrigation volume of 500 mL of physiological saline.^17^, ^18^ However, the irrigation volume required to completely eradicate disseminated malignant cells in CRC has not yet been determined. Previous studies have addressed appropriate irrigation solution volumes for patients with rectal cancer. Maeda et al. reported that 2000 mL of irrigation solution is necessary for ECC elimination.^10^ Similarly, other researchers have recommended 1500 mL of physiological saline to reduce the risk of local recurrence.^19^ Our research results indicate that after a 2000 mL intraluminal washout, positive ECCs were observed in 1.7% of patients with sigmoid colon cancer and 10.7% of patients with rectal cancer. This suggests that a minimum of 1000 mL intraluminal washout is necessary for sigmoid colon cancer and 2000 mL for rectal cancer to eliminate detached malignant cells.

Free malignant cells spread from the tumor into the colonic lumen. Therefore, the spread of malignant cells within the lumen is a matter of concern. In patients with larger tumors, tumor cells were assumed to be detected more frequently. The multivariate analysis in this study identified the tumor size at the time of the 2000 mL intraluminal washout as an independent risk factor (Tables 2, 3). This finding suggests the possibility of mechanical stimulation of the tumor during surgeries, such as contact and compression, in patients with larger tumors. Surgeries in the pelvic cavity beyond the tumor are required, particularly in patients with lower rectal cancer. Regarding the surgical approach, Hasegawa et al. reported that open surgery significantly increases the presence of ECCs in the bowel compared with laparoscopic surgery.^20^ Open surgery, considered more traumatic, may facilitate the spread of malignant cells into the bowel. In this study, laparoscopic and robotic techniques were predominantly used, accounting for the majority of surgeries, with only a small percentage (4.5%) being open surgeries. No statistically significant differences were observed in the prevalence of ECCs between these methods. To determine whether laparoscopic and robotic surgeries reduce ECCs, a randomized study with a sufficient sample size would be necessary. However, considering the significant international standardization and distinct advantages of laparoscopic and robotic surgeries, conducting such investigations may not be feasible. Additionally, positive ECCs did not show statistically significant differences in relation to other clinical and pathological factors such as tumor invasion depth.

Various irrigation solutions have been used for bowel cleansing. These include physiological saline, distilled water, cetrimide, chlorhexidine, povidone‐iodine solution, and ethanol.^21^, ^22^ In a study on bowel cleansing before colorectal anastomosis, the use of sodium hypochlorite and povidone‐iodine reduces bacterial counts in the rectal stump.^23^ However, the precise relationship between intestinal bacterial count and factors such as ECC count and local recurrence remains unclear. In this study, we investigated the effects of physiological saline and distilled water. Considering concerns regarding tissue damage associated with the concentration of agents in other irrigation solutions, further exploration is warranted. Physiological saline and distilled water are considered highly convenient because of their minimal tissue damage, cost‐effectiveness, and lack of the need for concentration adjustment. Additionally, our study demonstrated that the use of these irrigation solutions did not affect the positive rate of ECCs.

This study has several limitations. First, there is still insufficient evidence to demonstrate that the exfoliated cancer cells in the lumen actually cause local recurrence. Second, it focused solely on the volume and type of irrigation solution, which may not be representative of other facilities using different irrigation devices. Third, this was a single‐arm trial where the irrigation solution was changed during the study, and randomization was not performed. Finally, this was a small‐scale, single‐center prospective study. To validate these findings, larger prospective multicenter studies on a national scale, such as multicenter randomized controlled trials, are necessary.

In conclusion, in patients with sigmoid colon cancer with adequate preoperative bowel preparation, a long DM, and a small tumor size, a 1000 mL intraluminal washout may be sufficient. On the other hand, in patients with rectal cancer with a short DM and a large tumor size, a minimum of 2000 mL intraluminal washout is indicated.

## AUTHOR CONTRIBUTIONS

Shinji Furuya drafted the manuscript. Koichi Takiguchi, Hiroki Shimizu, Makoto Sudo, Shuguru Maruyama, Yuuki Nakata, and Yoshihiko Kawaguchi collected the intraluminal washout samples. Kunio Mochizuki and Tetsuo Kondo performed the cytological assessment of the washout fluid samples. Yuuki Nakata created the figures. Shinji Furuya and Daisuke Ichikawa conceived and designed the study and edited the manuscript. The final version of this manuscript has been approved by all authors.

## FUNDING INFORMATION

This research received no specific grant from any funding agency in the public, commercial, or not‐for‐profit sectors.

## CONFLICT OF INTEREST STATEMENT

Daisuke Ichikawa is Associate Editor of *Annals of Gastroenterological Surgery*. The remaining authors declare no conflict of interests for this article.

## ETHICS STATEMENT

Approval of the research protocol: Ethical approval was obtained from the University of Yamanashi Faculty of Medicine Ethics Committee for this prospective study using medical records (No. 1931). The protocol for this research project conforms to the provisions of the Declaration of Helsinki. All data were stored on a secure hospital server with access granted only to the authors of this study. Subsequent analyses were performed on the de‐identified datasets, and the database was accessed from July 2018 to December 2023. Patient privacy and confidentiality were maintained throughout all phases of the study.

Informed consent: Participants provided paper‐based informed consent at the time of admission, and their consent forms were digitized and stored in a database. Minors were excluded from the study.

Registry and the registration No. of the study/trial: N/A.

Animal studies: N/A.
